# Systems biology evaluation of cell-free amniotic fluid transcriptome of term and preterm infants to detect fetal maturity

**DOI:** 10.1186/s12920-015-0138-5

**Published:** 2015-10-22

**Authors:** Beena D. Kamath-Rayne, Yina Du, Maria Hughes, Erin A. Wagner, Louis J. Muglia, Emily A. DeFranco, Jeffrey A. Whitsett, Nathan Salomonis, Yan Xu

**Affiliations:** Perinatal Institute, Cincinnati Children’s Hospital Medical Center, Cincinnati, OH USA; Biomedical Informatics, Cincinnati Children’s Hospital Medical Center, Cincinnati, OH USA; Maternal-Fetal Medicine, University of Cincinnati College of Medicine, Cincinnati, OH USA

**Keywords:** Amniotic fluid, Fetal lung maturity, Transcriptome, Prenatal diagnosis

## Abstract

**Background:**

Amniotic fluid (AF) is a proximal fluid to the fetus containing higher amounts of cell-free fetal RNA/DNA than maternal serum, thereby making it a promising source for identifying novel biomarkers that predict fetal development and organ maturation. Our aim was to compare AF transcriptomic profiles at different time points in pregnancy to demonstrate unique genetic signatures that would serve as potential biomarkers indicative of fetal maturation.

**Methods:**

We isolated AF RNA from 16 women at different time points in pregnancy: 4 from 18 to 24 weeks, 6 from 34 to 36 weeks, and 6 from 39 to 40 weeks. RNA-sequencing was performed on cell-free RNA. Gene expression and splicing analyses were performed in conjunction with cell-type and pathway predictions.

**Results:**

Sample-level analysis at different time points in pregnancy demonstrated a strong correlation with cell types found in the intrauterine environment and fetal respiratory, digestive and external barrier tissues of the fetus, using high-confidence cellular molecular markers. While some RNAs and splice variants were present throughout pregnancy, many transcripts were uniquely expressed at different time points in pregnancy and associated with distinct neonatal co-morbidities (respiratory distress and gavage feeding), indicating fetal immaturity.

**Conclusion:**

The AF transcriptome exhibits unique cell/organ-selective expression patterns at different time points in pregnancy that can potentially identify fetal organ maturity and predict neonatal morbidity. Developing novel biomarkers indicative of the maturation of multiple organ systems can improve upon our current methods of fetal maturity testing which focus solely on the lung, and will better inform obstetrical decisions regarding delivery timing.

**Electronic supplementary material:**

The online version of this article (doi:10.1186/s12920-015-0138-5) contains supplementary material, which is available to authorized users.

## Background

Amniotic fluid (AF) is a dynamic mixture that both contributes to and reflects the status of the fetus [1–3], and has been shown to provide a screenshot into the maturational processes of fetal development [2–5]. At present, AF from different time points in pregnancy is used to provide obstetricians and pregnant women with important information for decision-making about pregnancy management and delivery planning, such as mid-trimester screening for aneuploidy, diagnostic testing for intra-amniotic infection, or fetal lung maturity testing [6–8]. However, with debate on the usefulness of fetal lung maturity testing [9], and the advent of non-invasive methods of prenatal diagnosis, practice patterns are changing to make amniocentesis, and thereby, analysis of AF, a much rarer occurrence [10, 11].

Ultimately, development of non-invasive methods for fetal testing, for example, by sampling maternal serum or urine, would minimize the use of invasive procedures such as amniocentesis. However, for discovery purposes, AF has important advantages over other maternal sourced specimens. AF contains larger amounts of fetal and pregnancy-related DNA, RNA, and proteins than maternal serum, particularly in the first and second trimesters of gestation, when most prenatal screening is performed [2, 12–14]. Most circulating fetal DNA fragments in maternal serum are short. Therefore, highly sensitive methods of detection are needed to distinguish the small size and quantity of fetal DNA from maternal DNA in maternal serum samples.

**Fig. 1:**
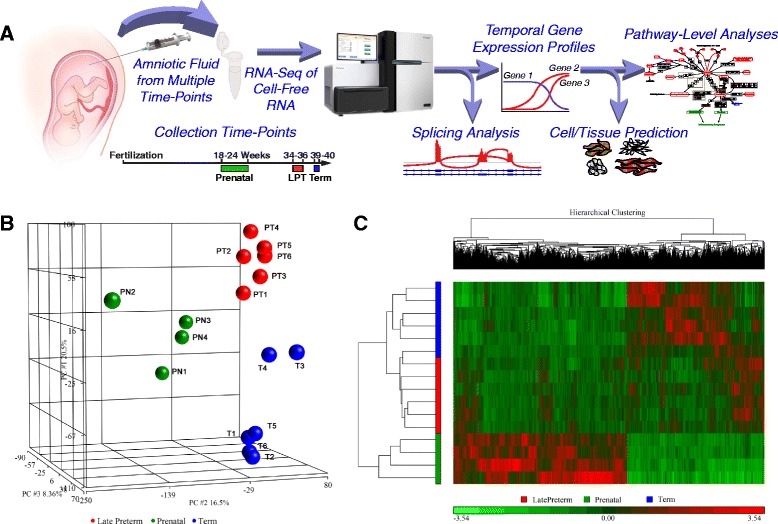
**a** Transcriptome analysis of RNAs isolated from AF at different gestational stages. RNAs isolated from 16 AF samples (4 Prenatal: 18-24 weeks, 6 Late Preterm: 34–36 weeks, 6 Term:39–40 weeks) were subjected to the AF transcriptome analysis. **b** Principal component analysis (PCA) of the top 3 identified components of RNA-seq samples showing the primary separation of samples by gestational ages. Prenatal (*Green*), Late Preterm (*Blue*) and Term (*Red*). **c** Heatmap produced by hierarchical clustering of genes and samples, showing the identification of differentially expressed genes in prenatal vs later preterm vs term AF samples

While cell-free fetal RNA and DNA in maternal serum can be used for prenatal screening, these analyses have not been extensively studied for the purpose of understanding the heterogeneous process of overall fetal maturation of multiple organ systems, with the goal of improving current methods of fetal maturity testing, which have focused primarily on the lung. We hypothesized that an unbiased study of AF cell-free RNA would enable identification of new biomarkers to assess multiple organ maturation in both term and preterm fetuses, and would include transcripts and alternative RNA isoforms that are uniquely expressed by the fetus. In the present study, we performed a small scale analysis, comparing RNA sequencing output from cell-free RNA from human AF obtained from three time points in pregnancy (prenatal 18–24 weeks, late preterm 34–36 weeks, and term 39–40 weeks) to determine if unique organ selective gene expression signatures are present that would be useful to assess fetal maturation (Fig. 1a).

## Methods

### Patient recruitment

The study was approved by the Institutional Review Boards at Cincinnati Children’s Hospital Medical Center, University of Cincinnati Medical Center, Good Samaritan Hospital, and The Christ Hospital in Cincinnati, Ohio. Written consent was obtained from study participants. Patients undergoing amniocentesis for prenatal diagnosis purposes consented to the acquisition of 10 mL of additional fluid to be banked and analyzed for our study. In addition, patients at any gestational age who were delivering by Cesarean section consented to the collection of 10 mL of AF after the uterine incision, and prior to rupture of the amniotic sac. Pregnant women who were delivering by Cesarean section also consented to data collection on their pregnancy, delivery, and clinical outcomes of their newborn infants.

For this small scale study, we selected 4 second trimester prenatal (PN) diagnosis samples (18–24 weeks), 6 late preterm (PT) samples (34–36 weeks), and 6 full term (FT) samples (39–40 weeks) from our AF biorepository, after excluding multiple gestation pregnancies, and pregnancies diagnosed with major congenital or chromosomal abnormalities. The prenatal diagnosis samples were obtained via amniocentesis. All the late preterm and term samples were obtained at the time of Cesarean section, except one late preterm sample and one term sample which were obtained via amniocentesis the day prior to delivery. All samples were pre-existing in an amniotic fluid biorepository. In selecting samples, we also attempted to ensure there would be samples from both male and female fetuses, and samples that had the neonatal morbidities of interest. Additional file 1: Table S1 indicates clinical data surrounding the pregnancy and newborn, including gestational age at which the samples were obtained, sex of the fetus, pregnancy complications (including pre-eclampsia, chorioamnionitis, and indication for Cesarean section), and the neonatal morbidities of respiratory insufficiency and gavage feeding. Four samples collected from term fetuses for pilot RNA isolation and sequencing purposes were de-identified, and not associated with clinical data.

### RNA isolation

All AF was immediately placed in AssayAssure tubes with standardized buffer. Samples were centrifuged, aliquotted and then stored at −80 °C until they were ready to be processed for RNA isolation. Approximately 6 mL of AF was used from each patient, and cell-free RNA was isolated from the supernatant of the fluid using the QIAamp Circulating Nucleic Acid Kit (QIAGEN), as has been previously described [15].

### RNA-sequencing analysis

RNA-sequencing was performed by the Cincinnati Children’s Hospital Sequencing Core, with a read-depth of 25–54 million reads per sample for 50 nt single-end reads (Additional file 1: Table S1). Two parallel analyses were performed to ensure validity and reproducibility of the results. The raw sequenced reads were aligned from FASTQ files to the human genome build GRCh37/hg19 and the UCSC reference transcriptome (https://ccb.jhu.edu/software/tophat/igenomes.shtml) using TopHat 2.0.9 and Bowtie2 with default parameters to identify both known and novel exons and junctions. Samples were further processed via Trimmed Mean normalization [16]. Adapters were retained in the reads as these improved overall the percentage of aligned reads (data not shown). All samples passed quality control assessment was performed using FASTQC and AltAnalyze. For differential expression analyses, an analysis of variance (ANOVA) was performed on AF RNA samples from each of the gestational stages, for genes with a reads per kilobase per million (RPKM) >1 in at least one sample. Differentially expressed genes were identified using one-way ANOVA followed by three paired comparison (i.e., PN vs. FT, PN vs. PT, and PT vs. FT). A gene is considered to be differentially expressed when a probability *P* value ≤0.05 (with FDR correction) and expression fold change ≥1.5 in at least one paired comparison. Comparison of specific preterm morbidities was performed using an moderated empirical Bayes *t*-test and fold >2 in AltAnalyze, due to the small number of samples in this cohort [17].

Differentially expressed genes were subject to Self-Organizing Maps clustering to identify gene clusters induced and suppressed with advanced gestational ages respectively. Gene-set enrichment analysis and comparison was performed using the software Toppgene (https://toppgene.cchmc.org/) and GO-Elite in AltAnalyze, where only terms with an FDR adjusted enrichment *p* <0.05 was considered for further evaluation. Raw and processed sequencing data have been deposited in GEO and SRA (http://www.ncbi.nlm.nih.gov/geo/query/acc.cgi?token=sjqpwsskhbchnsv&acc=GSE68180).

For alternative splicing analyses, junction.bed files were input in AltAnalyze to calculate percent spliced in (PSI) values for reciprocally expressed junctions from junction read counts, using annotations derived from Ensembl 72 and UCSC annotated mRNAs. This same analysis was performed on RNA-Seq junction reads from the Illumina Body Map project (http://www.ebi.ac.uk/arrayexpress/experiments/E-MTAB-513/). Read coverage plots were produced from Broad’s IGV Sashimi-Plot function (https://www.broadinstitute.org/igv/).

### AF tissue markers prediction

Two parallel tissue/cell-type prediction approaches were employed in these studies for independent verification. To evaluate time-point specific differences in cell and tissue markers, we used the LineageProfiler gene marker database (https://sourceforge.net/p/altanalyze/wiki/LineageProfiler/), derived from hundreds of distinct normal mouse and human cell and tissue sources in the software GO-Elite [18]. For these cell and tissue-prediction analyses, GO-Elite Fischer-Exact enrichment test *p* <0.05 was required for downstream analyses. To identify tissue/cell markers in independent AF samples at different pregnancy stages, we first filtered genes with expression level >90 percentile of all samples, and then we compared the abundantly expressed genes in AF from preterm, late preterm and full term samples to identify common vs unique expressed genes. We mapped the top 10 % highly expressed genes to tissues/cells using the gene expression atlas data downloaded from Genomics Institute of the Novartis Research Foundation (GNF) [19]. We defined a gene as enriched in tissue A if the average expression of the gene in tissue A was 3 times greater than its average expression in all other 66 tissues. We defined a tissue specific gene marker as the gene not only enriched in tissue A, but also expressed highest in tissue A and its expression in tissue A was at least 1.5 times higher than its expression in any other tissues.

## Results

### Transcriptomic profiles of amniotic fluid from different gestational stages

After passing quality control, samples were clearly separated by gestational age group of prenatal, preterm or term, as previously defined (Fig. 1b). Hierarchical clustering of differentially expressed genes segregated by gestational age revealed two major expression patterns: genes induced with time and genes suppressed with time (Fig. 1c). The changes between late preterm and full term fetuses are modest at a global level (PCA), but the two groups were readily separable when requiring False Discovery Tests *p* <0.05 as shown.

**Fig. 2:**
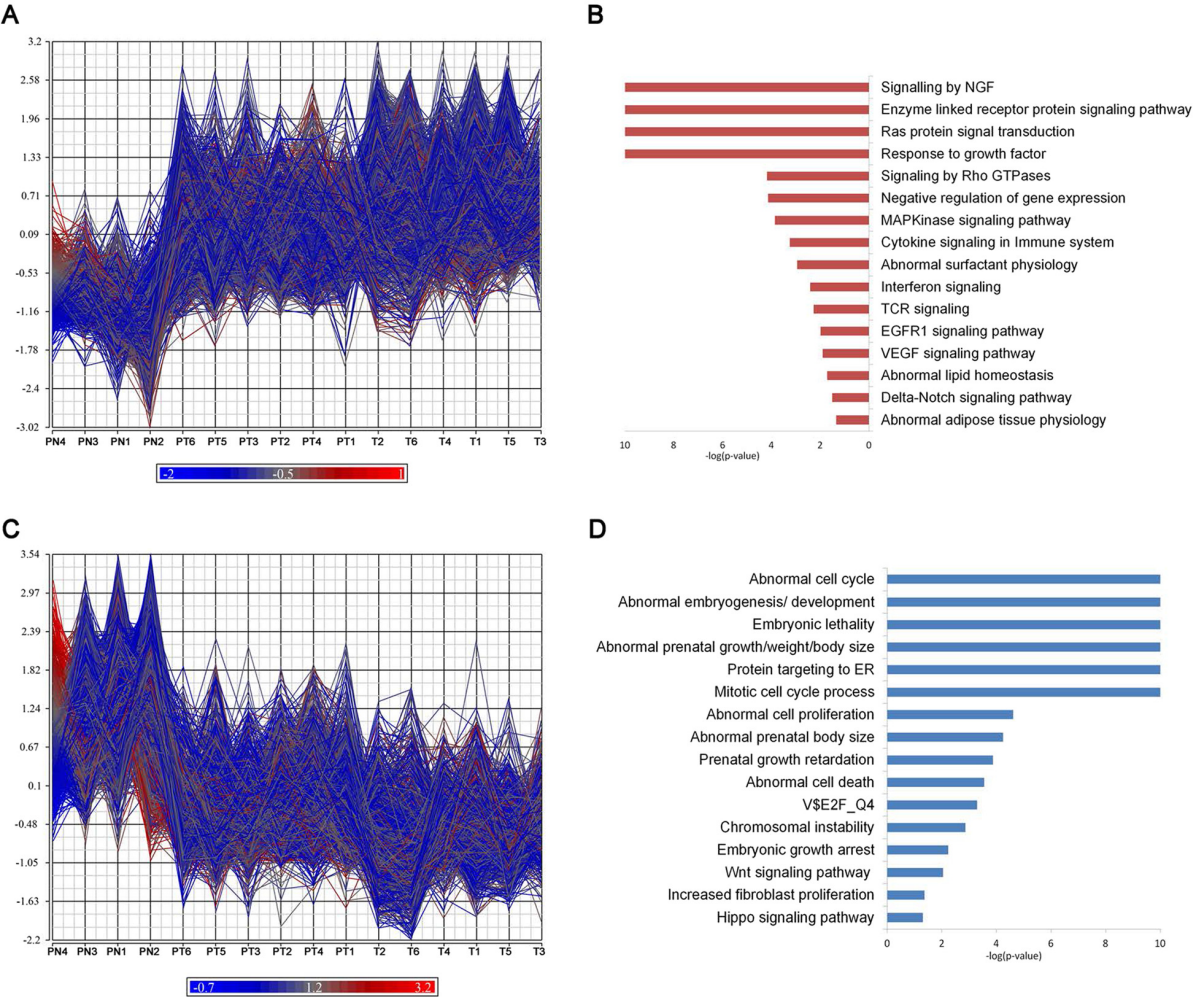
Identification of dynamic gene markers in AF samples. Genes with gestational time dependent increasing (**a**) or decreasing (**c**) trends were identified. Functional classification of these two classes of genes revealed distinctive enriched biological processes, pathways, mouse phenotypes and transcription factor binding sites (TFBS) associated these two groups of genes.Enriched functional classifications corresponding to genes that increase throughout gestation were displayed in panel (**b**), and enriched functional classifications corresponding to genes that decrease throughout gestation were displayed in panel (**d**). Gene set functional enrichment analysis was performed using Toppgene suite (https://toppgene.cchmc.org/)

**Fig. 3:**
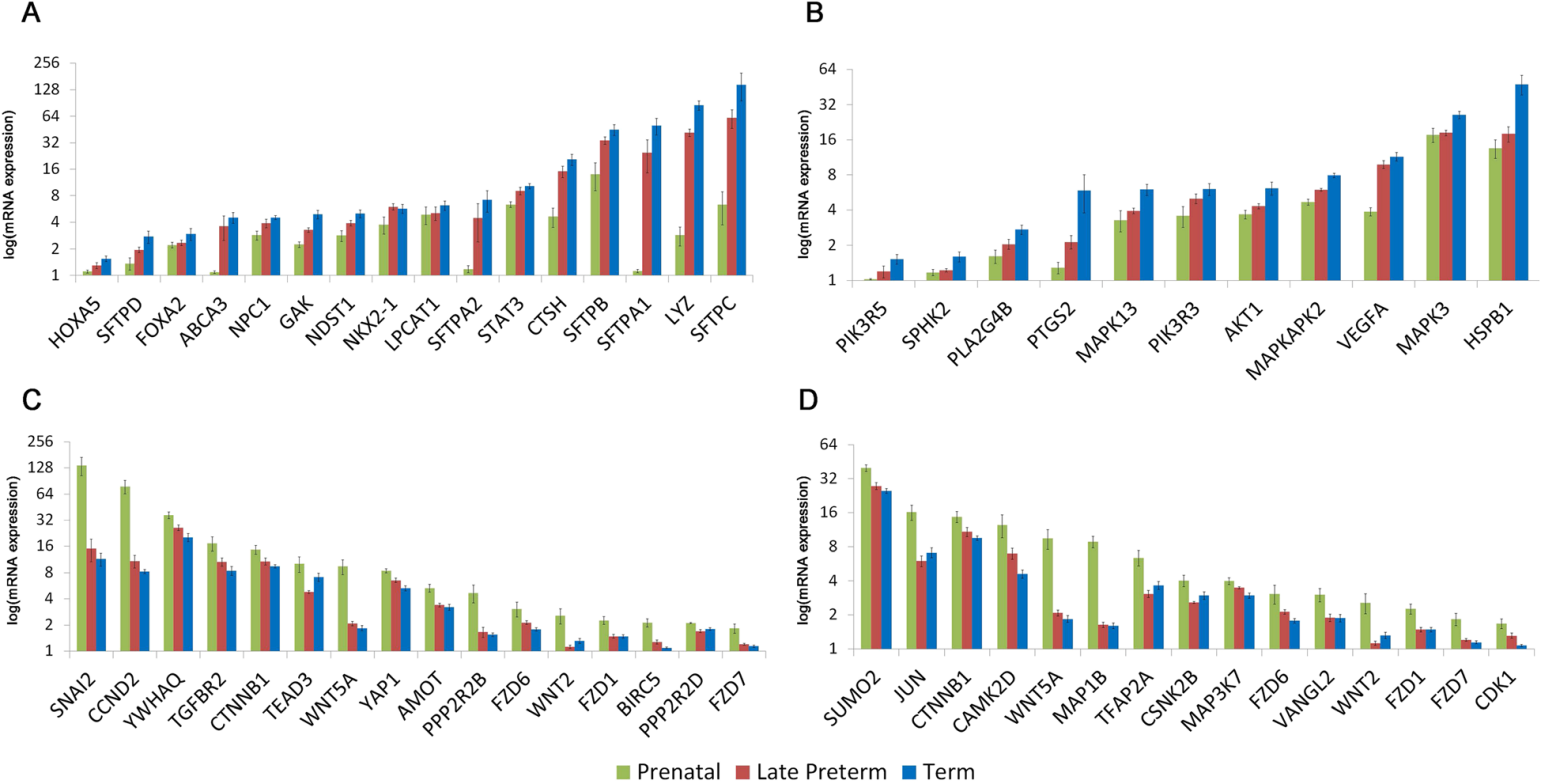
Dynamic change of expression patterns of genes regulating important pathways/processes in AF samples. Genes related with (**a**) surfactant physiology and (**b**) VEGF signaling were induced with increasing gestational ages while genes involved in (**c**) Hippo signaling and (**d**) Wnt Signaling had significantly suppressed expression with increasing gestational ages. Prenatal samples are noted in *Green*, Late Preterm in *Red*, and Term in *Blue*

Gene set functional enrichment analyses revealed distinctive biological processes, pathways and mouse phenotypes associated with the two major clusters (i.e., gene expression varying in a gestational time manner) (Fig. 2). Genes induced with advancing gestation included those that were functionally enriched in various signaling transduction pathways (NGF, RAS, MAPK, VEGF, EGFR1 signaling), lipid and surfactant homeostasis, mediated cell immune response and response to growth factors (Figs. 2b and 3a). Mutations or deletions of genes in this group are known to influence lipid homeostasis, surfactant physiology, and adipose and liver morphology. Genes whose mRNA abundance was inversely correlated with increased gestational ages were functionally enriched in cell cycle, protein targeting to ER, cell proliferation, embryogenesis and development (Fig. 2c, d). Mutations or deletions of genes in this group are associated with embryonic lethality in mouse models, abnormal embryogenesis/development, abnormal prenatal growth/body size, and embryonic growth arrest (Fig. 2d).

Genes related to surfactant physiology and VEGF signaling pathway were significantly increased in full term AF samples (Fig. 3). Genes regulating pulmonary surfactant function are important for lung function at birth; surfactant deficiency results in Respiratory Distress Syndrome (RDS) in premature infants. mRNAs encoding surfactant proteins (*SFTPA1*, *SFTPB*, *SFTPC*, and *SFTPD*) and those involved in lipid synthesis and processing (*LPCAT1*, *ABCA3*, *CTSH* and *LYZ*), and regulation (*FOXA2*, *NKX2-1*, *HOXA5*) were increased with advancing gestational age in AF (Fig. 3a). VEGF signaling was previously identified as a critical factor in perinatal lung function [20]; mice with defective VEGF die of respiratory failure at birth. Intra-amniotic or intra-tracheal delivery of VEGF improved surfactant production and protected preterm newborn mice from respiratory failure [20]. In the present study, key components in the VEGF signaling pathway including *VEGFA*, *AKT1*, *HSPB1*, *MAPK13*, *MAPK3*, *PIK3R3*, *PTGS2* and *SPHK2* were increased in full term AF (Fig. 3b).

In contrast to the induction of RNAs essential for lung development and differentiation, genes involved in the Wnt and Hippo signaling pathway were more highly expressed in prenatal AF samples. We observed expression of genes in the Wnt (*FZD1*, *FZD6*, *FZD7*, *JUN*, *PPP2R5E WNT2* and *WNT5A*) and Hippo signaling pathways (*AMOT*, *BIRC5*, *CCND2*, *CTNNB1*, *FZD1/6/7*, *PPP2R2B*, *PPP2R2D*, *SNAI2*, *TEAD3*, *TGFBR2*, *YAP1*, *YWHAQ*, Fig. 3c, d) decreased with advancing gestation. Both the Wnt and Hippo pathways are known to play important roles in morphogenesis, tissue growth and organ size [21]. The Hippo pathway regulates Wnt/beta-catenin signaling in coordinating morphogenetic signals with organ growth [22].

### Genes differentially expressed in full term versus late preterm AF

**Fig. 4:**
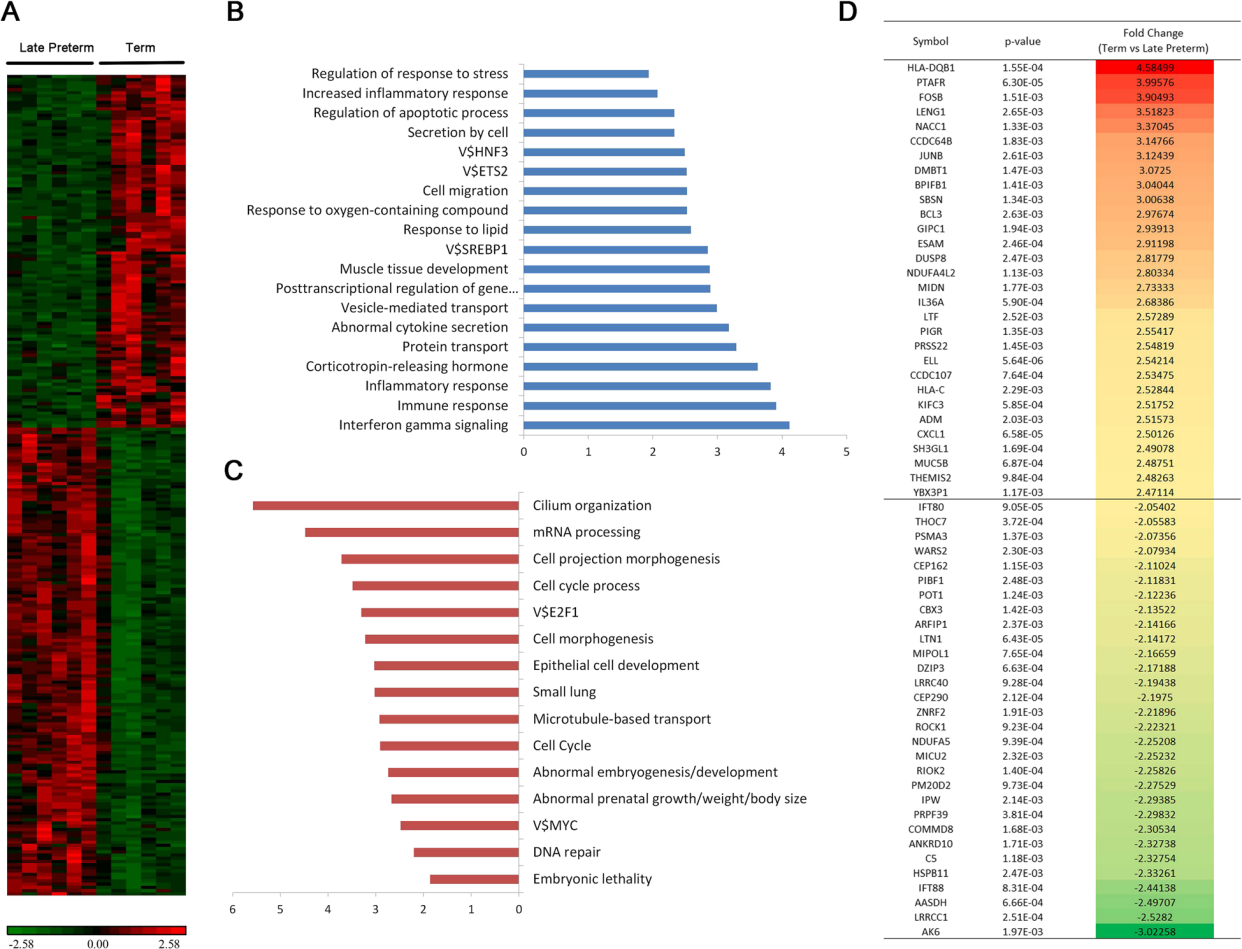
Differentially expressed genes in Term versus Late Preterm AF samples. **a** This heatmap depicts the 257 genes that were significantly differentially expressed in Late Preterm versus Term samples. **b**, **c** Functional enrichment analysis revealed distinct biological processes and pathways associated with genes differentially expressed in Late Preterm versus Term (**b** = enriched in full term infants, **c** = enriched in late preterm infants). **d** The top 60 significantly changed genes in Term versus Late Preterm. *P*-value is calculated by student *T*-test with Benjamini–Hochberg FDR correction (Benjamini Yoav, Hochberg Yosef. Controlling the false discovery rate: A practical and powerful approach to multiple testing. JRSS-B. 1995;57(1):289–300). Gene set functional enrichment analysis was performed using Toppgene suite (https://toppgene.cchmc.org/). The transcription factor notations reflect the type of position-specific scoring matrix used (e.g., V$ for vertebrate matrix library)

AF samples from late preterm (34–36 weeks, *N* = 6) and full term (39–40 weeks, *N* = 6) fetuses were compared to identify genes differentially expressed in the later stages of pregnancy because this is the period when fetal maturity testing would be most helpful for delivery planning. Two hundred fifty-seven genes were differentially expressed in late preterm versus full term fetuses; among these, 146 had higher expression in the late preterm period, where 111 were more highly expressed in term fetuses compared to late preterm fetuses. Processes such as “immune response”, “protein transport”, “response to stress/growth factor/lipid” and “apoptosis” were increased as assessed by Gene Ontology biological pathways in full term AF [23]. Processes including “cilium morphogenesis”, “mRNA processing”, “cell cycle”, and “protein catabolism”, were upregulated in late preterm fetuses in comparison with full term infants (Fig. 4b–c). Mutations or deletions of genes in these classes are known to associate with “small lung”, “decreased lean body mass” and “prenatal growth retardation”. Figure 4d lists the top 60 significantly induced (upper panel) or suppressed (bottom panel) genes in term versus late preterm fetuses, respectively.

### Identification of tissue/cell markers in AF collected at different stages of pregnancy

**Fig. 5:**
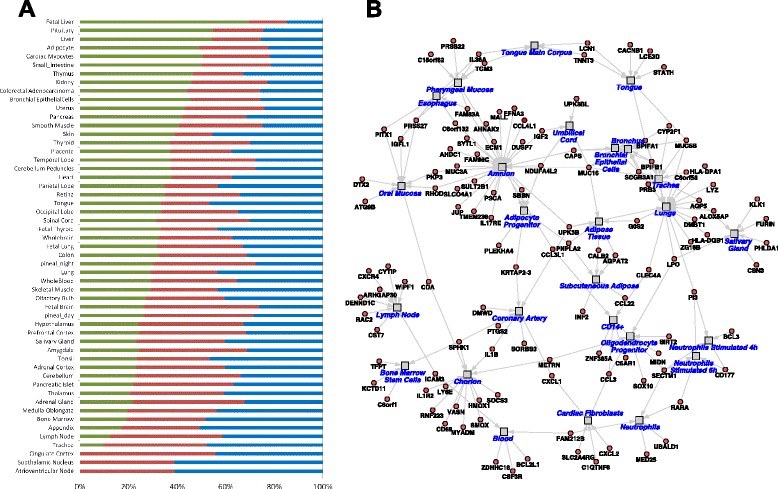
Tissue/cell markers in AF collected at different stages of pregnancy were identified. **a** Expression distribution analysis was used to identify genes expressed greater than 90 % quantile in Prenatal, Late Preterm or Term AF samples. Tissue specific gene markers were identified to meet two criteria: 1) the gene expression in tissue A should be at least 3 fold higher than its average expression across 66 tissues, and 2) the gene expression in tissue A should be at least 1.5 fold higher than any of the 66 tissues. Relative amount of tissue selective gene markers expressed in AF at the three gestational age periods were calculated and shown in bar graph. The portion of tissue specific genes in Prenatal samples is noted in *Green*, Late Preterm in *Red*, and Term in *Blue*. **b** Using the software GO-Elite, tissues and cell-types predicted to be enriched (Fisher’s Exact *p* <0.05) were identified and visualized as a network along with associated marker genes in AltAnalyze

To identify the tissue-specific gene expression patterns in AF, RNAs abundantly expressed in AF collected from each stage of pregnancy (>90 % in distribution analysis) were mapped to the GNF Gene Expression Atlas to estimate their relative expression across 66 different tissues (Fig. 5). The 16 AF samples expressed RNAs that can be directly traced to the fetal oropharynx and upper airway (trachea, tongue, salivary gland, tonsil), respiratory tract (lungs, bronchial epithelial cells), external barrier (skin, eye) and *in utero* environment (placenta, uterus), in addition to other major organ systems (brain, heart, liver, fat, kidney, pancreas, thyroid, thymus, intestine, bone marrow). Comparison of term infant AF and late preterm infant AF RNA-Seq profiles revealed hundreds of highly distinct transcripts that can be correlated with fetal organ maturity. These genes appear to largely reflect fetal as opposed to maternal RNAs, based on expression of the X-chromosome inactivation marker XIST and Y-chromosome specific markers (Additional file 2). As a cell-type prediction approach, we performed cell and tissue marker enrichment analysis in the software GO-Elite, which utilizes restricted tissue and cell-type (*n* = 300) specific markers (Fig. 5b). These results suggest that RNAs associated with neutrophils, lung, tongue, salivary gland, oral mucosa, adipocytes, oligodendrocyte progenitors and CD14+ cells, among others, are less highly expressed in late preterm compared to term fetuses. Highly specific cell/tissue markers, for example, for lung (*SCGB3A1*, *DMBT1*, *AQP5*), trachea (*BPIFB1*), salivary gland (*FURNIN*, *KLK1*, *LPO*), brain (*MIDN*, *METRN*) and neutrophil (*SECTM1*, *CD177*) were identified and associated with maturation. Our *in silico* cellular predictions system appears to identify cell selective markers that segregate fetuses based on organ maturity.

### Evaluation of late preterm co-morbidities

**Fig. 6:**
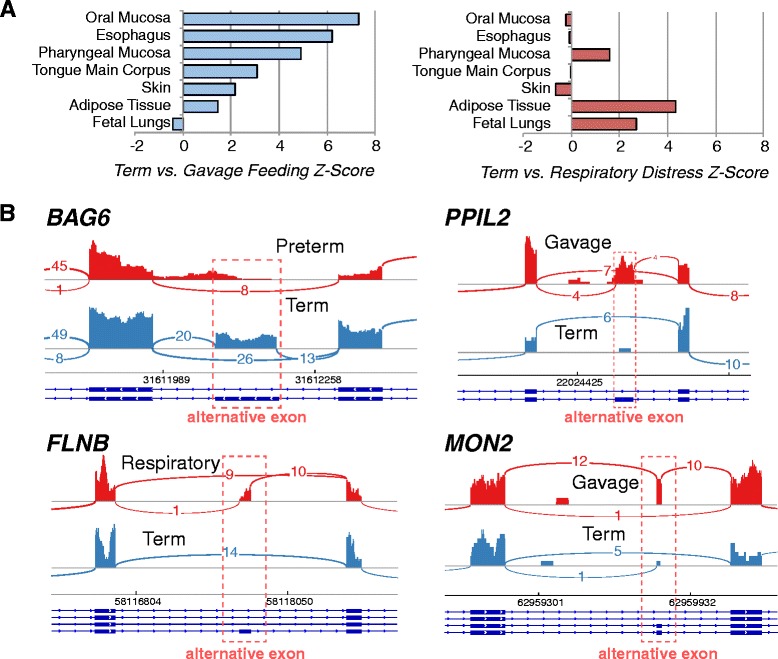
Implicated cells Systems, genes and isoforms with distinct preterm morbidities. **a** Tissue and cell-type enrichment analysis results from GO-Elite (enrichment z-scores) show differentially expressed genes from Term AF RNA-Seq samples compared to those Late Preterm infants that required gavage feeding (*Blue*) or had respiratory morbidity (*Red*). **b** Example selected splice variants with clearly observed alternative cassette-exon splicing events in four genes, displayed in the software IGV. Curved lines indicated exon-exon junction spanning reads, with the number of associated reads centered on the line. A representative sample from each indicated morbidity is shown in the IGV Sashimi-Plot. RefSeq mRNA exon transcript structures are shown below each plot

Multiple morbidities are frequently associated with preterm birth and result in clinically significant postnatal outcomes. Compared with term infants, late preterm infants have higher rates of premature neonatal morbidities, including need for respiratory support, gavage feeding, apnea/bradycardia of prematurity, problems with thermoregulation, and delays in neurodevelopment. Among the 6 late preterm infant samples analyzed, two infants required respiratory support, two required gavage feeding and one required both. As a proof of concept, we examined global changes in cell specific gene expression markers from these two small sample sets. Relative to term infants, late preterm infants requiring gavage feeding (*n* = 3) expressed fewer markers of salivary gland, oral mucosa, pharyngeal mucosa and tongue (Fig. 6a). On the other hand, infants that had respiratory insufficiency requiring respiratory support (*n* = 3) expressed similar percent of markers for salivary gland, oral mucosa, pharyngeal mucosa, and tongue, but did show a significant decrease in markers for adipose tissue and fetal lungs. This adipose tissue observation fits with prior observation that fat stores primarily increase in the third trimester of pregnancy. While cautious interpretation is needed due the small sample size of this cohort, these results are suggestive that AF biomarkers serve as indicators of organ system maturity. Analysis of splice variants expressed from late preterm infants requiring gavage feeding or respiratory support relative to term, found 78 and 66 regulated genes respectively, with only 18 shared between the two (Fig. 6b, Additional file 3). Among these splicing events, only half (74 out of 155), were found in a deeply sequenced panel of adult and placental tissues (Illumina Body Map2 dataset); thus, the remainder are likely to be fetal-specific (Additional file 4).

## Discussion

In 2008, the American College of Obstetricians and Gynecologists (ACOG) recommended fetal lung maturity testing for all patients born scheduled for elective delivery prior to 39 weeks gestation in order to avoid the consequences of respiratory distress from iatrogenic prematurity [24]. During that time, fetal lung maturity was often used as the sole criterion to establish that the infant was ready for postnatal life, while ignoring the potential immaturity of other organ systems. A growing body of evidence indicates that mature lung indices ascertained from AF do not spare a premature infant from other neonatal morbidities [6, 7, 25, 26], supporting the concept that fetal lung maturity testing is insufficient to determine readiness for postnatal life. As a result, ACOG recently published an updated practice guideline that fetal lung maturity testing was not useful to guide delivery timing in medically indicated preterm delivery [27]. However, some obstetricians still feel that such testing can be useful to weigh maternal and infant risks and benefits of early delivery [9, 28]. With the current debate, and the availability of improved genome-wide expression profiling methods, development of improved methods for determining fetal maturity are needed for delivery planning purposes, to better assess maternal/neonatal risks when planning for a preterm delivery.

The amniotic fluid transcriptome is a useful tool for providing insight into fetal development at different time points in pregnancy [5]. Previous studies have indicated that amniotic fluid supernatant provides a snapshot of developmental processes occurring in the fetus, and have unique gene expression patterns that are more fetal-specific compared to amniocytes [3]. Most of these studies have focused on the analysis of amniotic fluid supernatant from second trimester fetuses using microarray [2, 3] which have indicated a pattern of enrichment in brain-specific genes, also seen in our study (Fig. 5a). In addition, further studies have demonstrated a difference in gene expression patterns between AF obtained in the second trimester compared to that obtained at term [4].

Our present data build upon the existing literature and identifies unique gene expression patterns at different time points in pregnancy that could be utilized as biomarkers for a better understanding of overall fetal maturity. Our study is unique in the addition of samples from the late preterm period, which have not previously been examined in other studies, but provide a wealth of information about fetal development at times when obstetricians and patients are making decisions regarding delivery. The present work demonstrates the feasibility of AF transcriptomic profiles to study bioprocesses and pathways underlying fetal development. While the present sample size is small, the data identify biologically plausible candidate genes relevant to the maturation of multiple organ systems. The data are reassuring in that they demonstrate fetal lung maturation via surfactant-specific and lung morphogenesis-specific pathways with advancing gestational age, while also demonstrating maturation of other biological processes that indicate maturation of other organs. A comparison of late preterm infants with certain neonatal morbidities to term infants demonstrates that differences in gene expression could be ascertained to possibly assess neonatal risk for diverse morbidities. Such work aligns with the emphasis that multiple research agencies addressing the complex public health problem of preterm birth have placed on conducting research to identify biomarkers that could improve clinical risk assessment for preterm birth [29–31].

We recognize some limitations of this small study. Our study differs from previously published work because of the use of RNA-sequencing methodologies as opposed to microarrays, which has been shown to result in overlap in the most highly expressed genes compared to microarray, but may be more affected by technical variation [32]. Furthermore, our study does have a small sample size, and a lack of clinical data on four of our six term amniotic fluid samples. In addition, the circumstances under which the amniotic fluid was collected could potentially be attributed to certain pregnancy characteristics that could bias the results. In this present study, the majority of the late preterm deliveries were medically indicated due to pre-eclampsia, while the term deliveries were elective repeat Cesarean deliveries. Previous work by Edlow, *et al.* has demonstrated different gene expression patterns in pregnant obese women compared to those with normal body mass index [33], indicating that maternal clinical characteristics should be accounted for in future analyses. It is unclear whether amniotic fluid obtained from pregnancies where delivery was medically indicated would exhibit different patterns of fetal maturity from pregnancies in which amniotic fluid was sampled in the preterm period but the mother delivered at term. Our analysis remains pertinent for the situation where premature delivery of the infant is unavoidable or indicated, the more clinically relevant group to be studying in the first place. To continue on the path towards translating novel biomarkers into useful clinical tests, further validation and replication studies are needed, with larger sample sizes and multi-center confirmatory studies. These larger studies will need to account for possible confounding clinical variables that may affect how the amniotic fluid is obtained.

While it would be ideal to obtain amniotic fluid from the same pregnancy at different time points for comparison, this study design is neither practical nor feasible in real-life clinical settings. The ultimate goal is the development of less invasive prenatal testing that can be performed utilizing maternal serum or urine; with non-invasive prenatal diagnosis and changing patterns of amniocentesis for fetal lung maturity testing, amniocentesis is now a less common procedure in obstetrics. Given the advantages of amniotic fluid being less complex than serum and containing higher amounts of cell-free RNA and DNA that more directly reflect fetal status, analysis of the amniotic fluid transcriptome is a practical first step towards the biomarker discovery that can later be translated to less invasive methods. Such studies should ultimately include the analysis of fetal specific isoforms detected through deeper sequencing that might readily distinguish fetal from adult isoforms in peripheral maternal fluids, along with additional significant and difficult to diagnose prenatal and preterm conditions, for example, maternal-fetal infections, congenital malformations, or metabolic disorders. Such work will likely provide important insights into the simultaneous yet heterogeneous processes that contribute to fetal maturation, providing a broader view of maturation than our currently used fetal maturity tests, which focus solely on the lung.

## Conclusions

The present study represents a novel approach of dynamic RNA-seq profiling analysis of AF collected from three different gestational ages. Using both gene-based and tissue/cell-based approaches, we identified unique cell/organ-selective expression patterns and associated biomarkers (i.e., gene signatures) corresponding to different stages in pregnancy that can potentially identify fetal organ maturity and predict neonatal morbidity. Given the current debate about the usefulness of fetal lung maturity testing, this small study demonstrates the feasibility of using the amniotic fluid transcriptome to identify biomarkers for fetal organ maturation, and supports efforts to do a larger scale study in the future. Taking a broader overview of fetal maturity than just focusing on the lung will better enable obstetricians to make delivery planning decisions for preterm births, and prepare pediatricians and neonatologists for the various neonatal morbidities that these preterm infants may face.

## Availability of supporting data

Raw and processed sequencing data have been deposited in GEO and SRA (http://www.ncbi.nlm.nih.gov/geo/query/acc.cgi?token=sjqpwsskhbchnsv&acc=GSE68180).

## Additional files

Additional file 1: Table S1. Amniotic fluid samples and clinical characteristics. (DOCX 25.8 KB) Additional file 2:
**Detection of fetal specific sex chromosome associated gene expression in amniotic fluid RNA-Seq.** (PDF 358 kb) Additional file 3:
**Alternative exons detected through a reciprocal junction analysis of percent spliced in values from AltAnalyze among a panel of adult tissues and placenta by RNA-Seq.** (XLSX 110 kb) Additional file 4:
**Alternative exons in amniotic fluid detected through a reciprocal junction analysis of percent spliced in values from AltAnalyze between late preterm infants that required respiratory distress or gavage feeding compared to full term infants.** (XLSX 10303 kb)

## Abbreviations

AF: Amniotic fluid
ANOVA: Analysis of variance
RPKM: Reads per kilobase per million mapped reads
GEO: Gene expression omnibus
SRA: Sequence read archive
GNF: Genomics Institute of the Novartis Research Foundation
PCA: Principal component analysis
NGF: Nerve growth factor
RAS: An abbreviation of 'Rat sarcoma', reflecting the way the first members of the protein family were discovered. All Ras protein family members belong to a class of protein called small GTPase, and are involved in cellular signal transduction.
MAPK: Mitogen-activated protein kinases
VEGF: Vascular endothelial growth factor
EGFR: Epidermal growth factor receptor
ER: Endoplasmic reticulum
RDS: Respiratory distress syndrome
WNT: Wingless-type MMTV integration site family
HIPPO: The Hippo signaling pathway takes its name from one of its key signaling components—the protein kinase Hippo (Hpo). Mutations in this gene lead to tissue overgrowth, or a “hippopotamus”-like phenotype.
ACOG: American College of Obstetricians and Gynecologists
