# Supplementary material for: Systems biology evaluation of cell-free amniotic fluid transcriptome of term and preterm infants to detect fetal maturity
Source: BMC Med Genomics. 2015 Oct 22;8:67. doi: 10.1186/s12920-015-0138-5 (PMC4619218; doi:10.1186/s12920-015-0138-5)
Supplement: Additional file 1: Table S1. — Amniotic fluid samples and clinical characteristics. (DOCX 25.8 KB) [file 12920_2015_138_MOESM1_ESM.docx]

| **Table 1: Amniotic fluid samples and clinical characteristics** | | | | | | |  |  |  |  |  |  |  |  |
| --- | --- | --- | --- | --- | --- | --- | --- | --- | --- | --- | --- | --- | --- | --- |
| Sample | Alias | GA | Fetal/ | Known fetal complications | Indication for Cesarean section | Maternal HTN | PPROM | Chorio | Other pregnancy complications | Infant | Infant | Aligned RNA-Seq Reads | Total RNA-Seq Reads |  |
|  |  |  | infant sex |  |  |  |  |  |  | respiratory support | gavage |  |  |  |
|  |  |  |  |  |  |  |  |  |  |  | feeding |  |  |  |
| PN1 | PN1 | 19 3/7 | F | None |  |  |  |  | Elevated AFP |  |  | 25038352 | 36238973 |  |
| PN2 | PN2 | 18 5/7 | M | Echogenic intracardiac focus |  |  |  |  | None |  |  | 21006908 | 28406319 |  |
| PN3 | PN3 | 24 3/7 | F | None |  |  |  |  | Abnormal quad screen |  |  | 20821412 | 24292807 |  |
| PN4 | PN4 | 23 0/7 | M | Echogenic intracardiac focus |  |  |  |  | Isoimmunization |  |  | 18038813 | 20787072 |  |
| PT1 | PT5 | 35 0/7 | F | None | Pre-eclampsia | Yes | No | No | Gestational diabetes | Yes | No | 24512698 | 27777614 |  |
| PT2 | PT6 | 34 5/7 | M | None | Pre-eclampsia, breech | Yes | No | No | None | No | Yes | 26815616 | 31442678 |  |
| PT3 | PT7 | 35 2/7 | M | None | Placenta previa | Yes | No | No | None | Yes | No | 39069846 | 53639458 |  |
| PT4 | PT8 | 34 0/7 | F | None | Maternal cervical mass | Yes | No | No | Sample collected day prior to delivery for amnio dye procedure | Yes | Yes | 20338772 | 23359775 |  |
| PT5 | PT9 | 36 6/7 | M | None | Breech | Yes | No | No | Class H diabetes, intrauterine growth restriction | No | Yes | 25887465 | 32878186 |  |
|  |  |  |  |  | NRFHTs |  |  |  |  |  |  |  |  |  |
| PT6 | PT10 | 35 6/7 | M | None | Repeat | Yes | No | No | None | No | No | 25803786 | 33389779 |  |
| T1 | E2 | ≥39 0/7 | M |  |  |  |  |  |  |  |  | 35007426 | 50860442 |  |
| T2 | F2 | ≥39 0/7 | M |  |  |  |  |  |  |  |  | 25390653 | 50860442 |  |
| T3 | T3 | 39 | F | None | Repeat | No | No | No | No | No | No | 25790710 | 35007608 |  |
| T4 | T4 | 39 3/7 | F | None | Repeat | No | No | No | Sample collected day prior to delivery for fetal lung maturity testing | No | No | 21883068 | 29382946 |  |
| T5 | T1-5 | ≥39 0/7 | M |  |  |  |  |  |  |  |  | 23557882 | 34657095 |  |
| T6 | G2 | ≥39 0/7 | M |  |  |  |  |  |  |  |  | 18681915 | 26404593 |  |
