# Supplementary figures and images for: Systems biology evaluation of cell-free amniotic fluid transcriptome of term and preterm infants to detect fetal maturity
Source: BMC Med Genomics. 2015 Oct 22;8:67. doi: 10.1186/s12920-015-0138-5 (PMC4619218; doi:10.1186/s12920-015-0138-5)

# EIF1AY

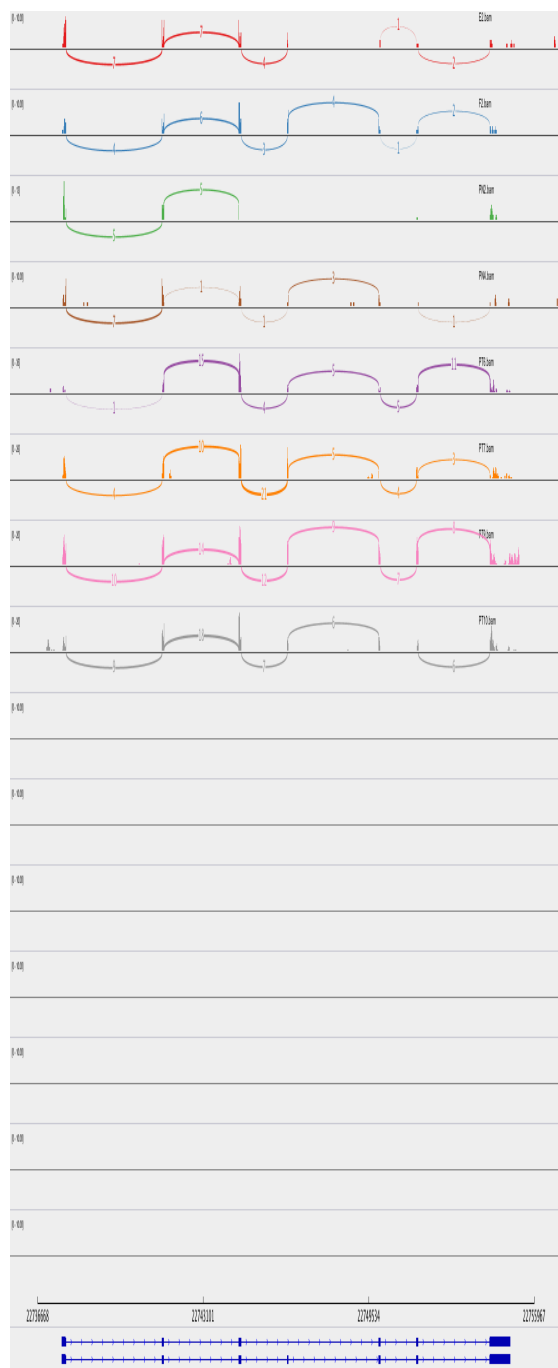

# XIST

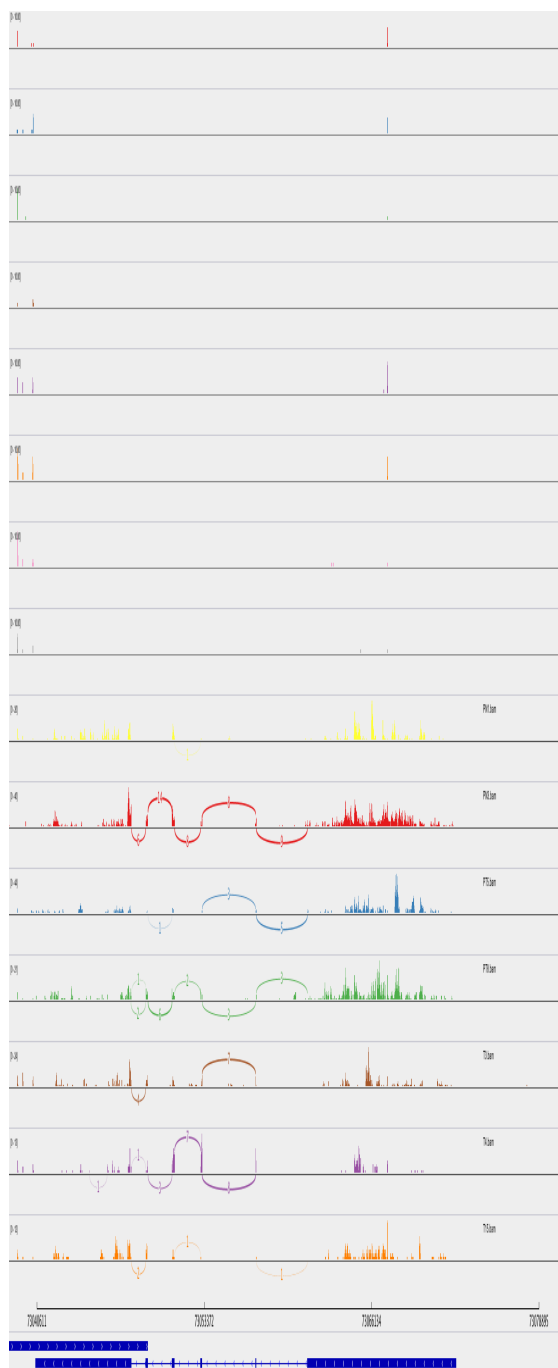

Male

Female

Supplement: Additional file 2: — Detection of fetal specific sex chromosome associated gene expression in amniotic fluid RNA-Seq. (PDF 358 kb) [file 12920_2015_138_MOESM2_ESM.pdf]
